# Extraosseous Ewing's sarcoma/peripheral primitive neuroectodermal tumour of the kidney: a case report and literature review

**DOI:** 10.1186/s12894-022-01146-w

**Published:** 2022-11-30

**Authors:** Jing Li, Fang Nie, Yan Li

**Affiliations:** Ultrasound Medicine Center, Lanzhou University Second Hospital, Chengguan District, Lanzhou, 730030 Gansu China

**Keywords:** Extraosseous Ewing's sarcoma/peripheral primitive neuroectodermal tumour, Nephrectomy, Contrast-enhanced ultrasound, Case report

## Abstract

**Background:**

Extraosseous Ewing's sarcoma/peripheral primitive neuroectodermal tumours(EWS/pPNETs) of the kidney are rare. Signs and symptoms are atypical in EWS patients. Presenting symptoms include haematuria, abdominal pain, or a palpable mass. A comprehensive review of the literature shows that it is difficult to make an accurate diagnosis based on physical examination alone. The imaging findings of EWS/pPNETs are nonspecific. We used contrast-enhanced ultrasound (CEUS) to diagnose an EWS/pPNET in our patient, which had never been reported previously to our knowledge.

**Case presentation:**

This article reports the case of a 20-year-old female with an abdominal mass and gross haematuria for 1 month. The ultrasound revealed a hypoechoic mass with a clear margin at the lower pole in the left kidney. CEUS demonstrated signs of annular enhancement and heterogeneous enhancement of the tumour, and simultaneous wash-in was predominant. Computed tomography images showed an elliptical low-density tumour. The patient underwent a left kidney resection, and the pathological diagnosis was an EWS/pPNET. Twenty-one days after the kidney operation, the patient underwent 8 cycles of a CAV (vinorelbine, ifosfamide, epirubicin) + IE (isocyclophosphamide, etoposide) chemotherapy regimen. Subsequently, radiotherapy (dose: 45 Gy, radiation field:the tumour bed following surgical resection) was administered for nearly 30 days. The patient had no signs of local recurrence or metastasis within a follow-up of 4 years.

**Conclusions:**

As a radiation-free, inexpensive, convenient, and repeatable examination method, ultrasound was the primary choice for kidney examination. Early CEUS was helpful to make an accurate diagnosis. Surgery and adjuvant radiation or chemotherapy administered in a timely manner can prevent further deterioration.

**Supplementary Information:**

The online version contains supplementary material available at 10.1186/s12894-022-01146-w.

## Background

Ewing's family of tumours (ESFTs), the rarest of oncologic disorders [1], are highly malignant tumours of soft tissue origin. ESFTs are common in adolescents and young adults and occur primarily in the bones or soft tissues of the limbs and rarely in the internal organs [2–4]. ESFTs include Ewing's sarcoma (ES), extraosseous sarcoma, and primitive neuroectodermal tumours (PNETs) [1]. The origin of these tumours is unclear, but they appear to be derived from cells migrating from the neural tube, with different ectodermal or neuronal differentiation abilities [5]. Histologically, ES/PNETs consist of a single circle of small cells, forming Homer Wright rosettes [6]. Primary Ewing's sarcomas of the kidney (ESKs) account for less than 1% of all renal tumours [7]. The clinical symptoms of ESKs are atypical and resemble renal colic symptoms, including an abdominal mass, abdominal pain, and haematuria [8]. More than 65% of ESK patients have distant metastases, and the common sites of metastases include the regional lymph nodes, lungs, and liver, of which the lungs are the most common site. The overall survival rate of patients is meagre, and most patients die from metastatic lung carcinoma [9, 10]. Since the first case was reported in 1975 by Seemayer et al. [11], there has been increasing interest in ESKs. However, there is still no clear understanding of this disease at present. To our knowledge, most researchers have reported their clinical symptoms, computed tomography (CT) or magnetic resonance imaging (MRI) manifestations, and treatment methods; however, few researchers have analysed the ultrasound appearance of ESKs. In this article, we summarize a patient's symptoms, imaging findings, and treatment method, as well as the findings of previously published studies. The investigators would like to discuss and share ultrasound findings that are consistently found in ESKs as well as criteria for developing imaging and treatment guidelines for ESKs.

## Case presentation

**Fig. 1:**
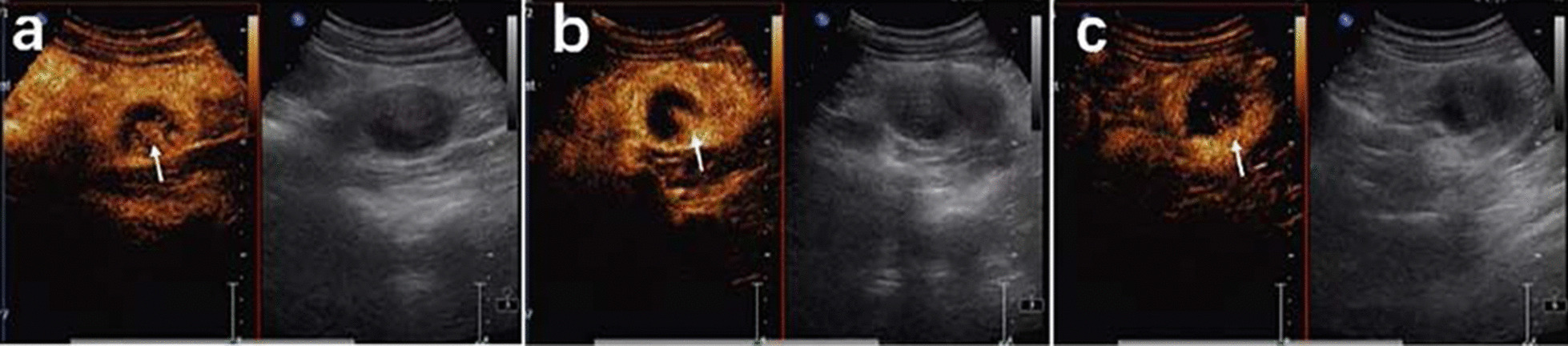
CEUS patterns of EWS/pPNET of the kidney in a 20-year-old female patient. **a** The lesion demonstrates annular enhancement in the cortical phase (arrows). **b** The lesion showed heterogeneous enhancement in the parenchymal phase (arrow). **c** The lesion demonstrates simultaneous wash-in with surrounding renal parenchyma in the late enhancement phase (arrow)

**Fig. 2:**
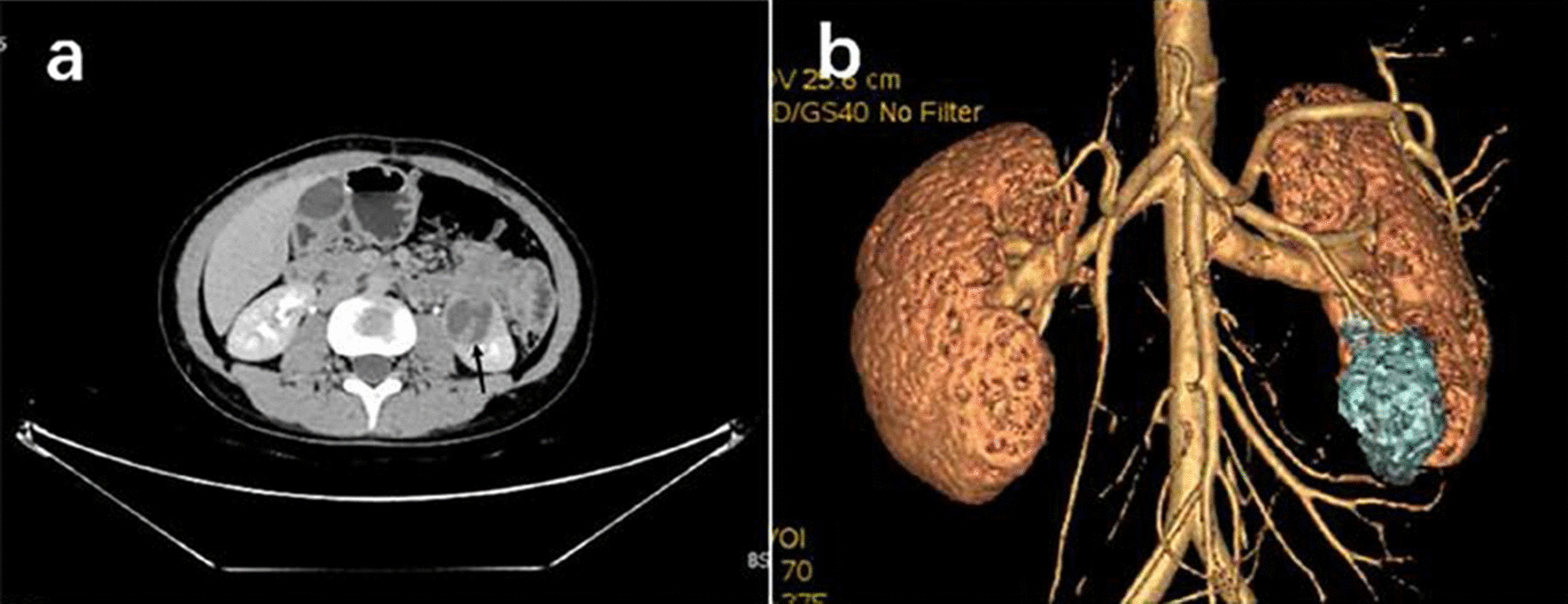
**a** Computerized tomography (CT)of the urinary system showed a heterogeneous mass in the left kidney(arrow). **b** Three-dimensional reconstruction of the kidney showed the lower 1/3 of the left kidney was occupied

**Fig. 3:**
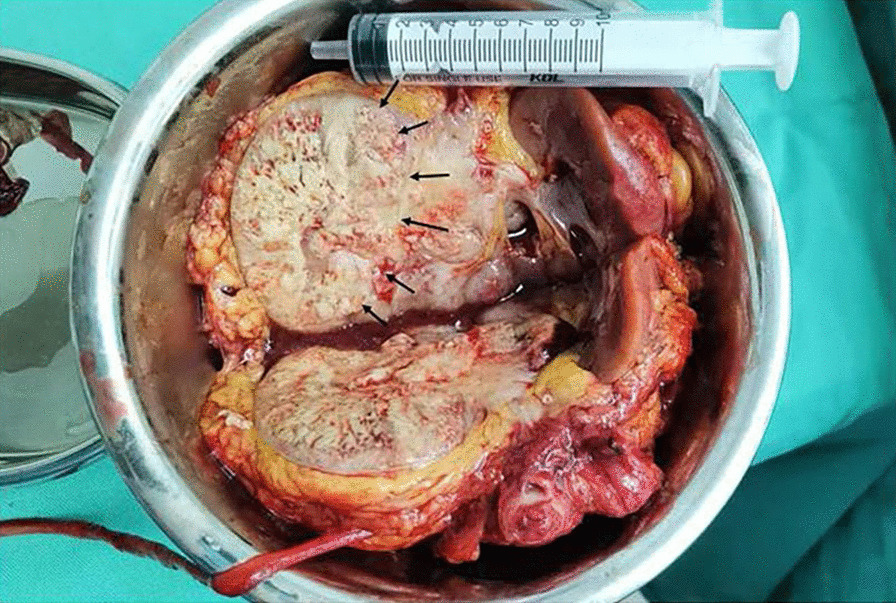
Postoperative gross pathology (left kidney): Gross total removal of the kidney was achieved. The tumor was a well-defined gray-white nodular mass with necrosis(arrow)

**Fig. 4:**
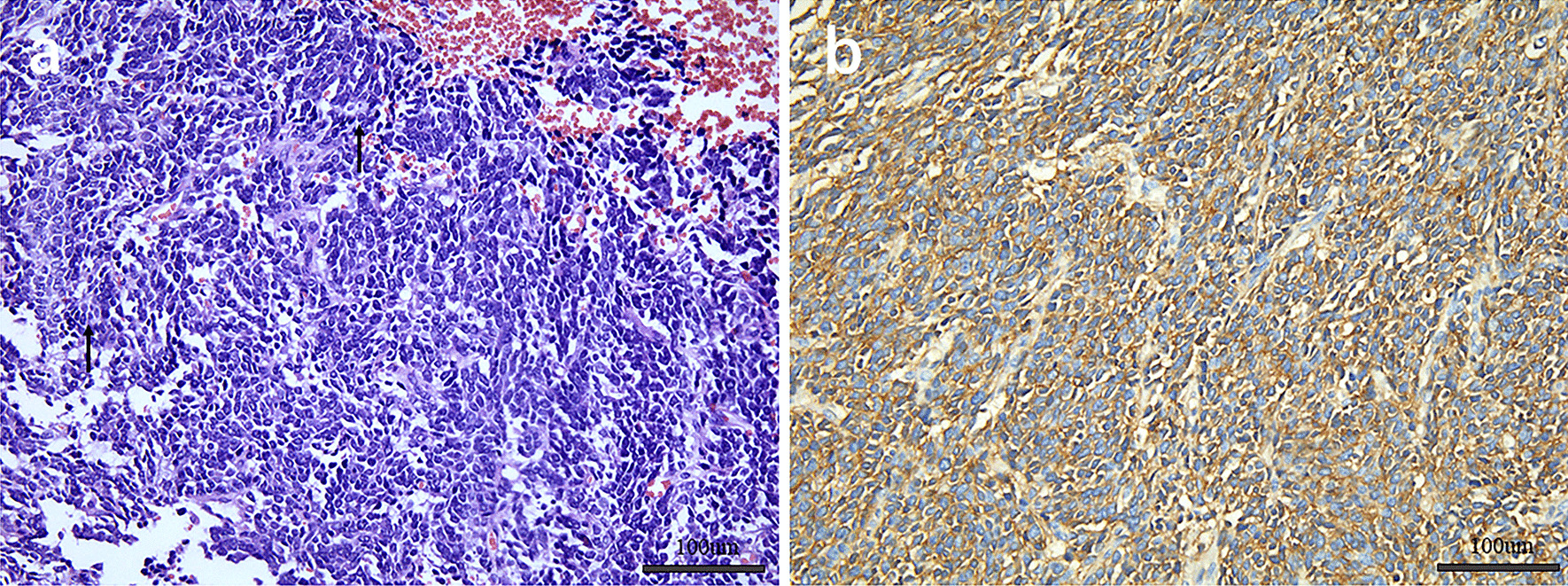
HE view suggests that the tumor cells were small, round, short spindle shape, densely arranged. The structure of the Homer right rosettes (arrow)can be seen. **b** Tumor cells showed positive immunoreactivity for CD99 (Fiqure 4 was acquired by Leica LAS X. The measured resolution was 1920*1440. We enhanced the resolution of the image to 300dpi (3862*1497) by PS(Adobe Photoshop 2020) in order to conform to standards of journal.）

A 20-year-old female presented to the hospital with an abdominal mass and gross haematuria for 1 month. She recently complained of left abdominal pain, nausea and vomiting, and noticeable weight loss. The patient did not have a family history of malignant tumours. The physical examination revealed abdominal distention with a large mass in the left upper quadrant. The mass was irregular, hard, immovable, nontender and without overlying skin changes. The laboratory test results for tumour markers were as follows: carbohydrate antigen 125 (CA-125), 136.89 U/mL (reference range: 0.00–35.00 U/ml); the levels of other tumour markers, such as carcinoembryonic antigen (CEA), carbohydrate antigen 199 (CA-199), and alpha-fetoprotein (AFP), were normal. Urine analysis showed that the red blood cell (RBC) count was 56/µl (reference range: 0.0–25.0), and the white blood cell (WBC) count was 59/µl (reference range: 0.0–25.0). Liver and kidney function test results and the complete blood count were within normal levels. Conventional ultrasound (US) examination showed a 4.5 × 3.2 cm irregular lesion at the lower pole of the left kidney. To further clarify the diagnosis of the tumour, contrast-enhanced ultrasound (CEUS) was performed, which presented signs of annular enhancement and heterogeneous enhancement of the tumour, and simultaneous wash-in was predominant. The possibility of a tumour lesion was considered (Fig. 1). A CT scan showed a 3.7 × 3.8 × 4.0 cm heterogeneous mass in the left kidney, which had blurry edges and a high-density dissepiment in the interior. Three-dimensional reconstruction of the kidney showed that the lower one-third of the left kidney was occupied (Fig. 2). Gross total removal of the tumour was achieved. The tumour was a 3.5 × 3 × 2.6 cm well-defined grey‒white nodular mass with necrosis (Fig. 3). Hematoxylin–eosin staining (HE) and immunohistochemistry (Leica DM4 B, DFC7000 T camera and LAS X software) were performed. Under the microscope, the tumour cells were small, round, short spindle-shaped, and densely arranged; Homer Wright rosettes were found; the cytoplasm of the tumour cells was sparse; and the nuclei were slightly enlarged and hyperchromatic. The tumour tissue was accompanied by extensive haemorrhage and necrosis. Immunohistochemistry showed the following: CD99 ( +), Vimentin ( +), EMA (−), CD10 (−), CD56 (−), syn (−), and NSE (−) (Fig. 4). Other immunohistochemistry staining see supplementary information (Additional file 1). The pathological diagnosis was an EWS/pPNET that did not invade the ureters. Subsequently, 21 days after the operation, the patient received 8 cycles of a CAV (vinorelbine, ifosfamide, epirubicin) + IE (isocyclophosphamide, etoposide) regimen from December 1, 2017, to May 11, 2018. Adjuvant radiotherapy (dose: 45 Gy, radiation field: the tumour bed following surgical resection) was also administered from June 11, 2018, to July 13, 2018. At the same time, the serum CA-125 level of the patient showed a gradual downwards trend after surgery and chemotherapy. The serum CA-125 levels returned to normal at the end of chemotherapy and radiotherapy. The patient had no signs of local recurrence or metastasis on CT scan within a follow-up of 4 years.

## Discussion and conclusions

EWS/pPNETS of the kidney are sporadically seen in clinical practice. The main pathogenesis is not clear thus far. Eighty to ninety-five percent of EWS/pPNET patients exhibit the chromosomal translocation T (11; 22) (q24; Q12), and 5–20% of patients often present with mutations in the EWS-ETS gene [9–13]. A comprehensive review of the literature shows that it is difficult to make an accurate diagnosis based on physical examination alone. The imaging findings of EWS/pPNETs are nonspecific. We used contrast-enhanced ultrasound to make a diagnosis of EWS/pPNETs in our patient, which had never been reported previously to our knowledge.EWS/pPNETs of the kidney are more common in male adolescents, with an average age of 29 years, and the male:female sex incidence ratio ranges from 2:1 to 3:1 [14]. The clinical symptoms and imaging findings are nonspecific. The clinical symptoms of EWS/pPNETs are atypical and resemble renal colic symptoms, including an abdominal mass, abdominal pain, and haematuria [8]. More than 65% of ESK patients have distant metastases, and the common sites of metastases include the regional lymph nodes, lungs, and liver, of which the lungs are the most common site. The overall survival rate of patients is meagre, and most patients die from metastatic carcinoma of lung [9, 10].

- Imaging methods, such as CT and MRI, have their own specific indications [14] to can help make a correct diagnosis [15]. The EWS/pPNET of the kidney was an inhomogeneous mass with unobvious renal vessels, no signs of invasion, and no calcifications on CT [16, 17]. In a 60-year-old patient with an EWS/pPNET of the kidney, ultrasound revealed an exophytic cortical cyst of the left kidney with irregular echogenic septa. Abdominal MRI and CT scans revealed a large lesion with necrosis of the mass. MRI showed homogeneous hypointensity on T1-weighted images and hyperintensity on T2-weighted images [18]. According to previous literature reports [19–23], we concluded that the CT characteristics of an EWS/pPNET of the kidney are as follows: (1) a large soft tissue mass, (2) the mass can be well defined, (3) necrosis can be found, (4) the renal vein or inferior vena cava may be involved, and (5) calcification is rare. Areas of high density correspond to areas of internal haemorrhage, and areas of low density correspond to areas of necrosis. The CT findings of the patient whose case is presented here are consistent with those previously reported [16–23]. As a radiation-free, inexpensive, and convenient examination method, US can be the primary choice in the diagnosis of EWS/pPNETs. Conventional US may fail to differentiate cystic and necrotic areas due to factors such as resolution. However, CEUS can solve this problem. The EWS/pPNET of the kidney mainly manifested as annular enhancement and heterogeneous enhancement on CEUS, and simultaneous wash-in was predominant in the EWS/pPNET of the kidney. Other common renal malignancies, such as clear cell renal cell carcinomas (ccRCCs), papillary renal cell carcinomas (pRCCs), and chromophobe renal cell carcinomas (chRCCs), can be differentiated from EWS/pPNETs of the kidney on CEUS. ccRCCs are rich in blood vessels, and the vessels of ccRCCs are large, irregular and distorted, with arteriovenous fistulas, which lead to the characteristics of early wash-in and hyperenhancement on CEUS [24]. Furthermore, the rapid tumour growth and proneness to ischaemic necrosis of ccRCCs lead to heterogeneous enhancement. In contrast, pRCCs and chRCCs, owing to the relative lack of vessels or the thick walls of vessels, often show hypoenhancement on CEUS. Previous studies [25] have reported that chRCCs mainly demonstrated simultaneous wash-in, while pRCCs mainly demonstrated slow wash-in. For the wash-out pattern, rapid wash-out mostly appeared in pRCCs and chRCCs. Additionally, EWS/pPNETs need to be distinguished from other uncommon renal tumours, such as adult Wilms, tumours, rhabdoid tumours, and renal clear cell sarcomas. Preoperative diagnosis of these tumours is difficult because there are no specific radiographic findings, and diagnosis relies primarily on histopathology.

Immunohistochemical and molecular diagnostic methods are critical to diagnose EWS/pPNETs. Pathological examination showed that the tumour cells were small, round, short spindle-shaped, and densely arranged. Homer Wright rosettes were found. Immunohistochemical results showed that the glycoprotein CD99 was expressed on the cell surface. The chromosomal translocation T (11;22) (q24; Q12) can be observed in 95% of EWS/pPNETs. However, despite the availability of immunohistochemical and molecular diagnostic methods, these tumours were also misdiagnosed on biopsy [2, 26, 27].

Currently, there is no universally accepted treatment regimens for EWS/pPNETs of the kidney based on treatment guidelines [28, 29], and the best treatment and natural history are unknown. The National Comprehensive Cancer Network (NCCN) and the European Society of Medical Oncology [28, 29] recommend that the current treatment of EWS/pPNETs of the kidney be based on the treatment experience of other ESFTs, which include radical nephrectomy combined with chemotherapy and adjuvant radiotherapy [10, 28, 30, 31]. The most commonly used chemotherapy regimen is CAV/IE or VACD/IE [5]. In the Grier study [32], alternating VACD or VACD/IE was used for nonmetastatic EWS/pPNET patients, which significantly improved the overall survival (72% vs. 61%) of those patients. The VDC/IE alternating cycle did not improve the outcome of patients with metastatic disease. Approximately 30–40% of Ewing’s sarcoma patients will relapse [5], and the following regimen has been proposed: alternating ifosfamide with etoposide and carboplatin, ifosfamide with etoposide, docetaxel with gemcitabine, and temozolomide with irinotecan [33–36]. The determination of the chemotherapy regimen for the patient whose case is presented here was based on the situation of the patient, who was without metastasis or recurrence. The prognosis for EWS/pPNET patients with active treatment remains objective. The 4-year overall survival was 85% for patients without metastasis and 47% for patients in whom the tumour had invaded the renal vein or inferior vena cava or who had distant metastasis [37]. The overall survival rate of EWS/pPNET patients is higher than that of Ewing's sarcoma of the bone (ESB) patients [38, 39]. If no metastasis is found after the end of chemotherapy for an EWS/pPNET of the kidney, the 5-year and 10-year survival rates are significantly increased to 70% and 60%, respectively [21, 40–42].

In conclusion, EWS/pPNETs of the kidney are very rare tumours that can be diagnosed by CEUS. However, multimodal imaging combined with pathological examination is necessary. Total surgical resection and auxiliary treatment can improve the prognosis of patients with EWS/pPNETs.

## Supplementary Information

**Additional file 1**. Supplement of immunohistochemical staining.

## Data Availability

All data and figure generated or analyzed during this study are included in this published article.

## Abbreviations

EWS/pPNET: Ewing's sarcoma/peripheral primitive neuroectodermal tumour
CEUS: Contrast-enhanced ultrasound
CT: Computed tomography
ESFT: Ewing's family of tumours
ES: Ewing's sarcoma
PNET: Primitive neuroectodermal tumour
ESK: Ewing's sarcoma of the kidney
MRI: Magnetic resonance imaging
CA-125: Carbohydrate antigen 125
CEA: Carcinoembryonic antigen
CA-199: Carbohydrate antigen 199
AFP: Alpha-fetoprotein
RBC: Red blood cells
WBC: White blood cells
US: Ultrasound
HE: Hematoxylin–eosin staining
ccRCC: Clear cell renal cell carcinoma
pRCC: Papillary renal cell carcinoma,
chRCC: Chromophore renal cell carcinoma
ESB: Ewing's sarcoma of the bone
