# Supplementary material for: Extraosseous Ewing's sarcoma/peripheral primitive neuroectodermal tumour of the kidney: a case report and literature review
Source: BMC Urol. 2022 Nov 30;22:197. doi: 10.1186/s12894-022-01146-w (PMC9710127; doi:10.1186/s12894-022-01146-w)
Supplement: Supplementary file 1 — Additional file 1. Supplement of immunohistochemical staining. [file 12894_2022_1146_MOESM1_ESM.docx]

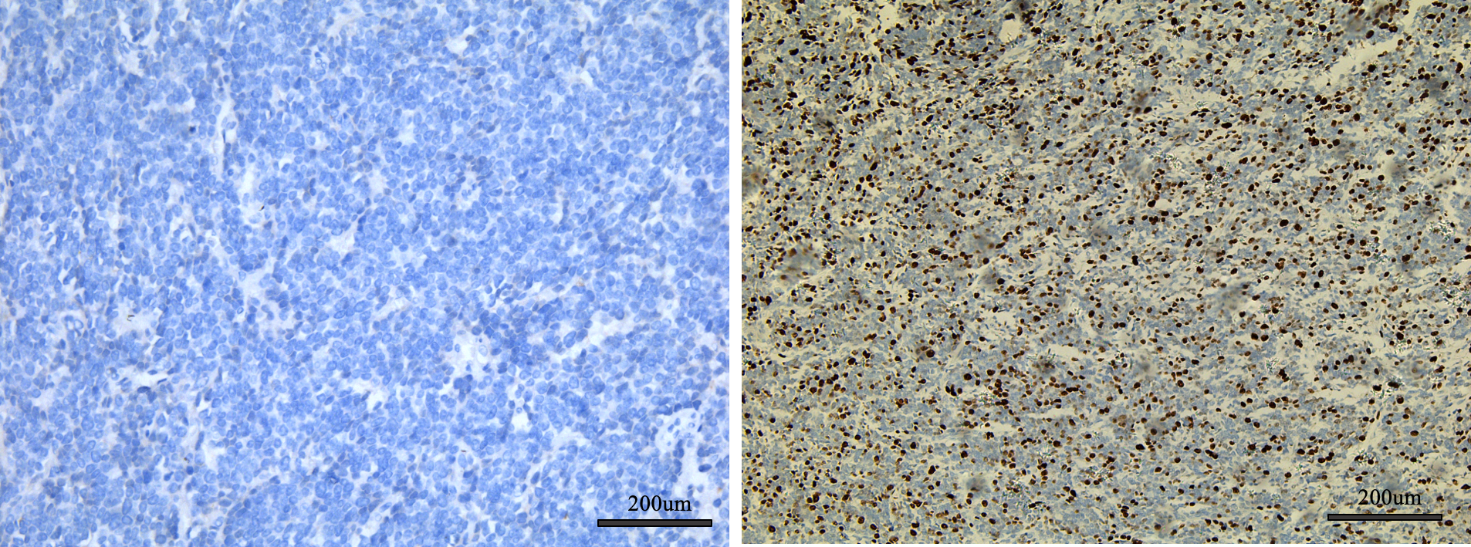
Immunohistochemistry suggested CD56(-)、Ki67≥50%


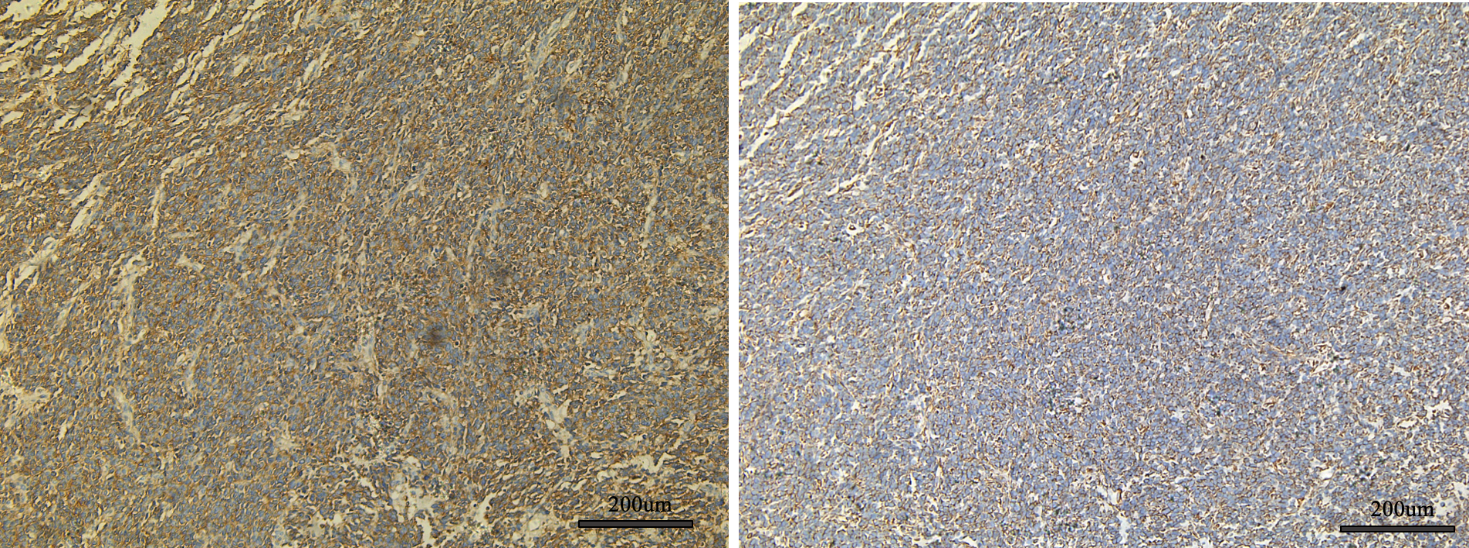


Immunohistochemistry suggested CD99(+)、Vim(+)


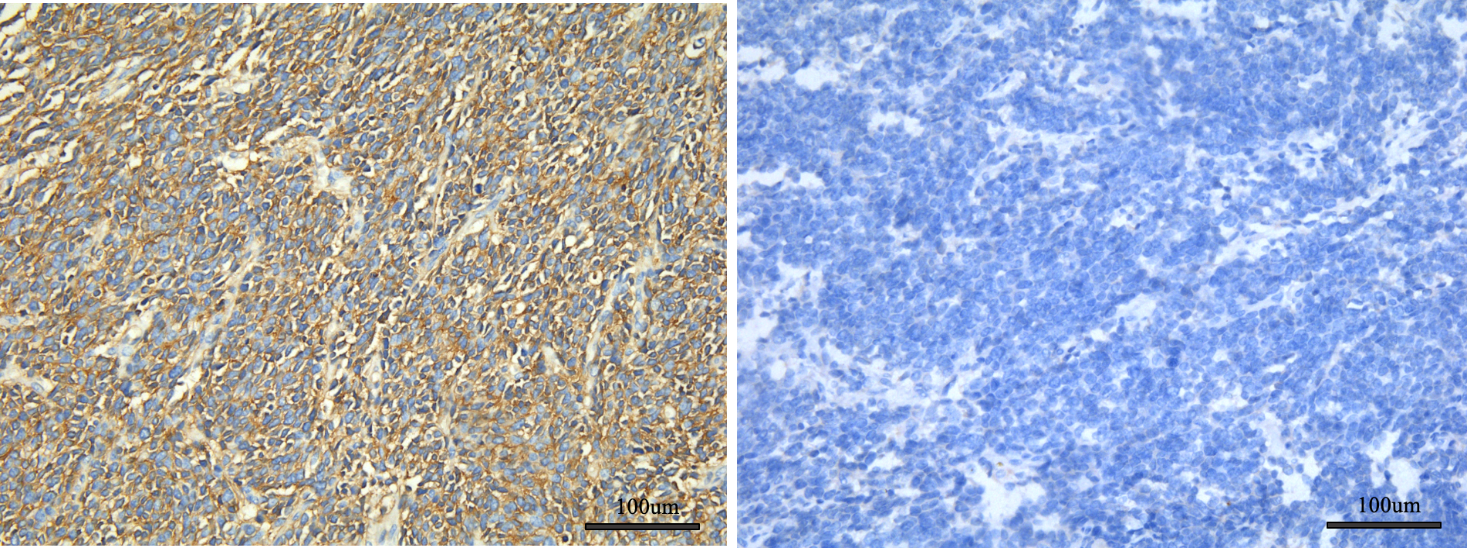


Immunohistochemistry suggested CD99(+)、CgA(-)


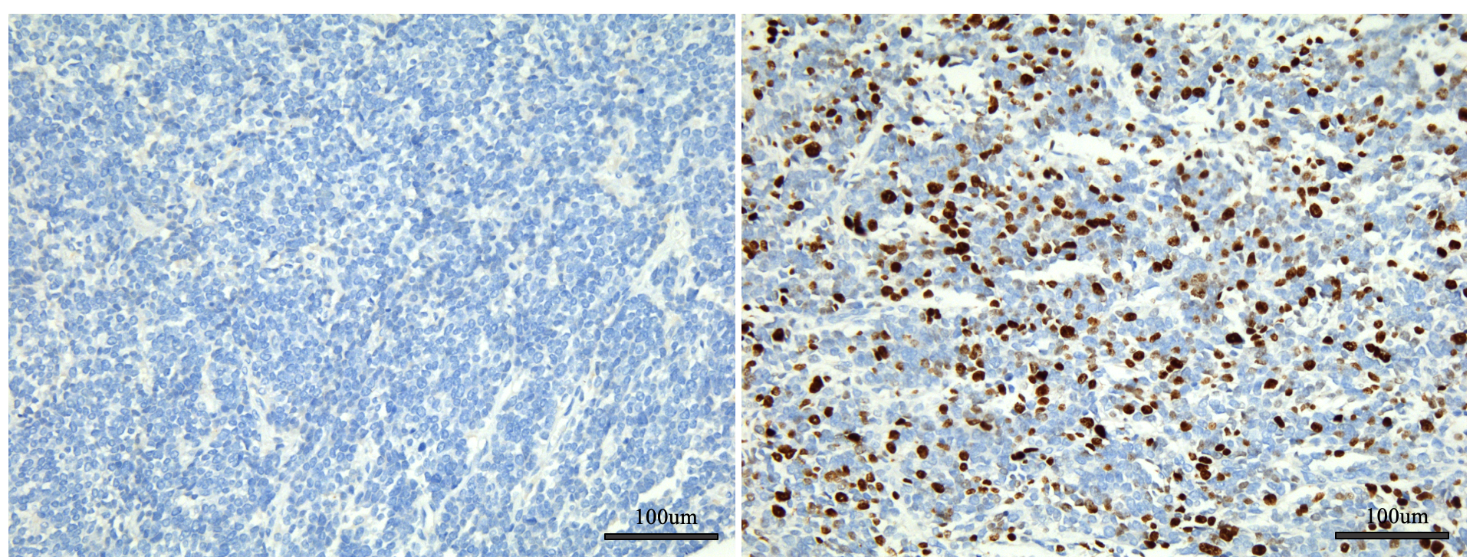


Immunohistochemistry suggested EMA (-)、Ki67≥50%


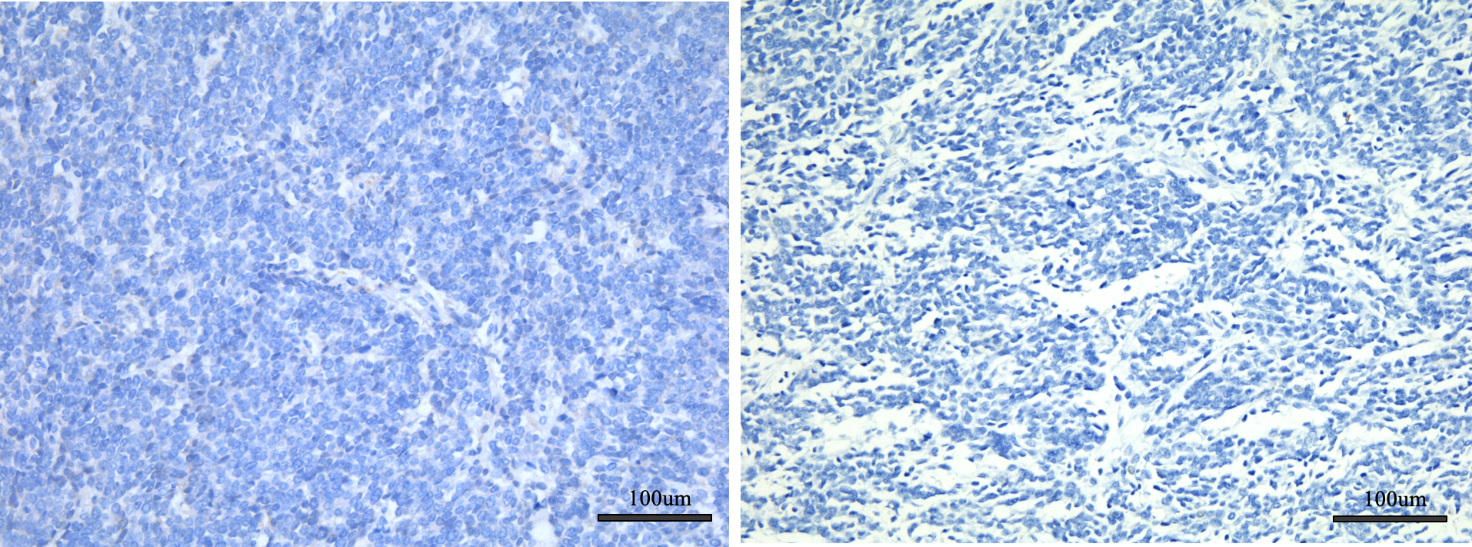


Immunohistochemistry suggested NSE (-)、S-100(-)


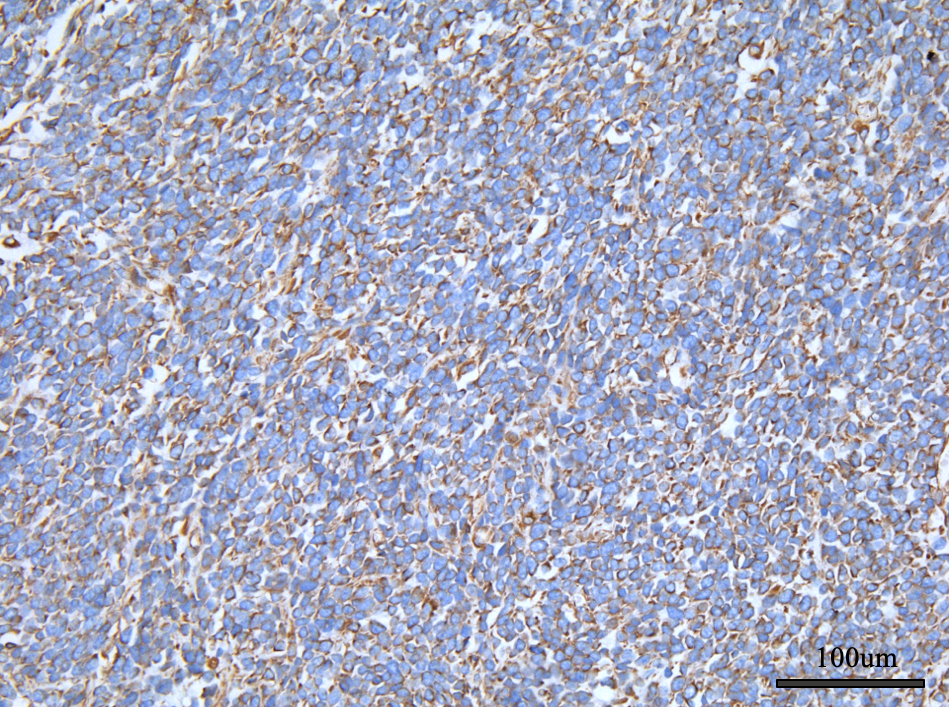


Immunohistochemistry suggested Vim (+)


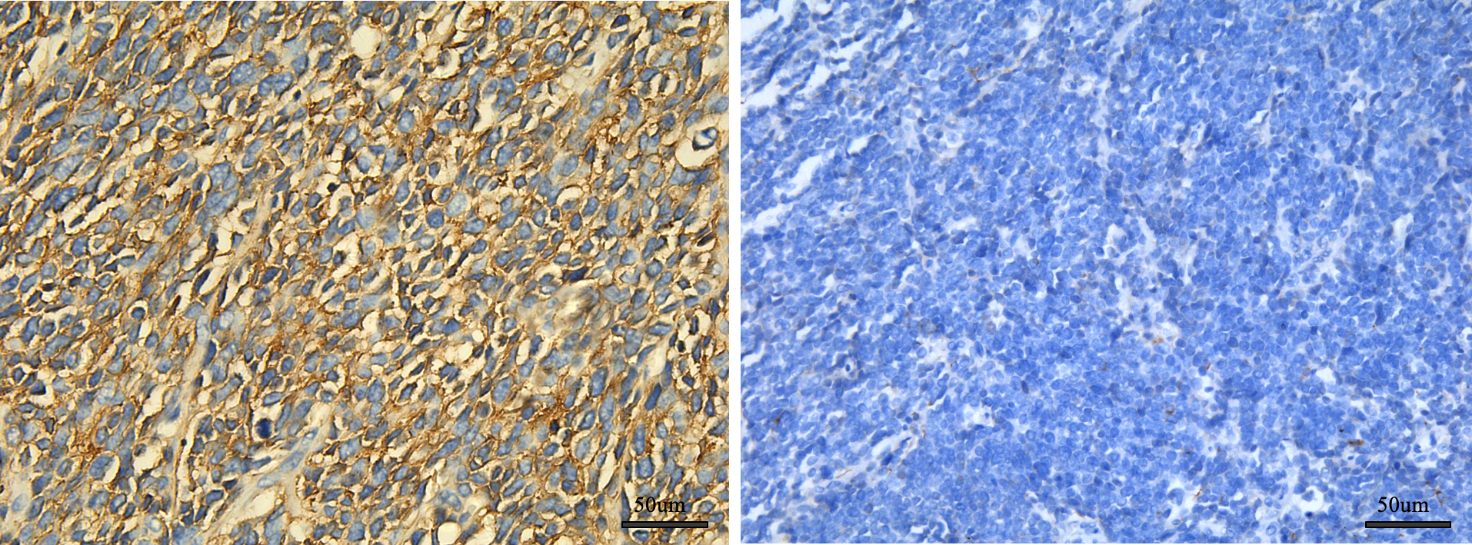


Immunohistochemistry suggested CD99(+)、CK2(-)


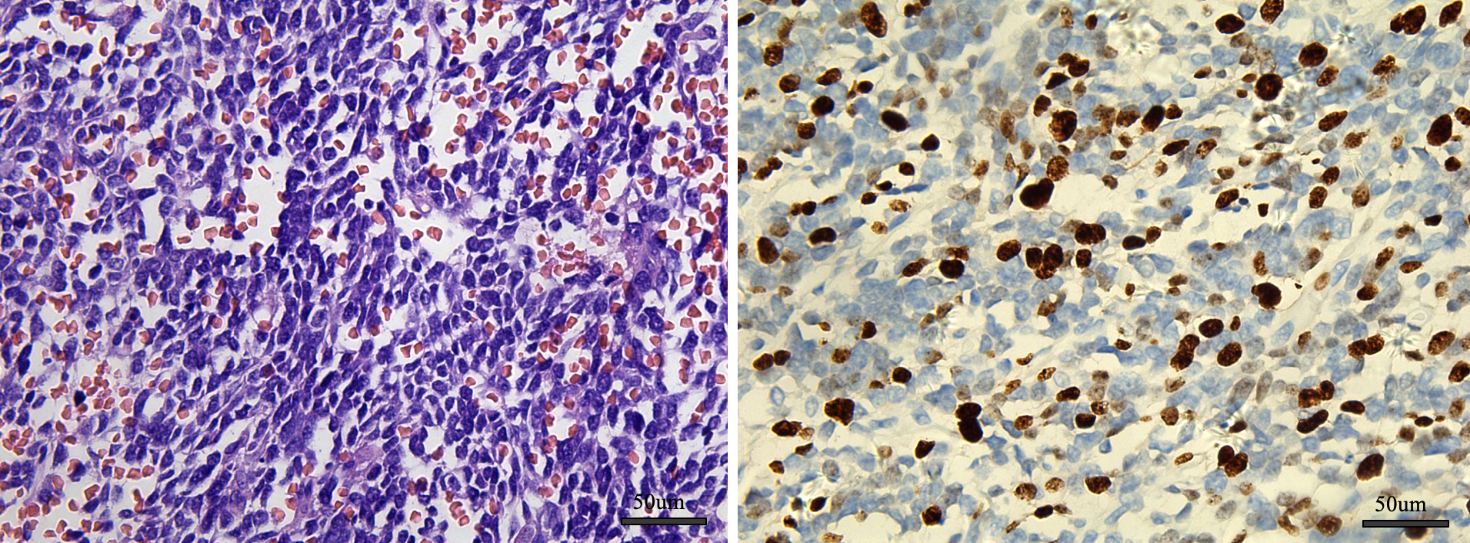


HE X400 Immunohistochemistry suggested Ki67（+++）


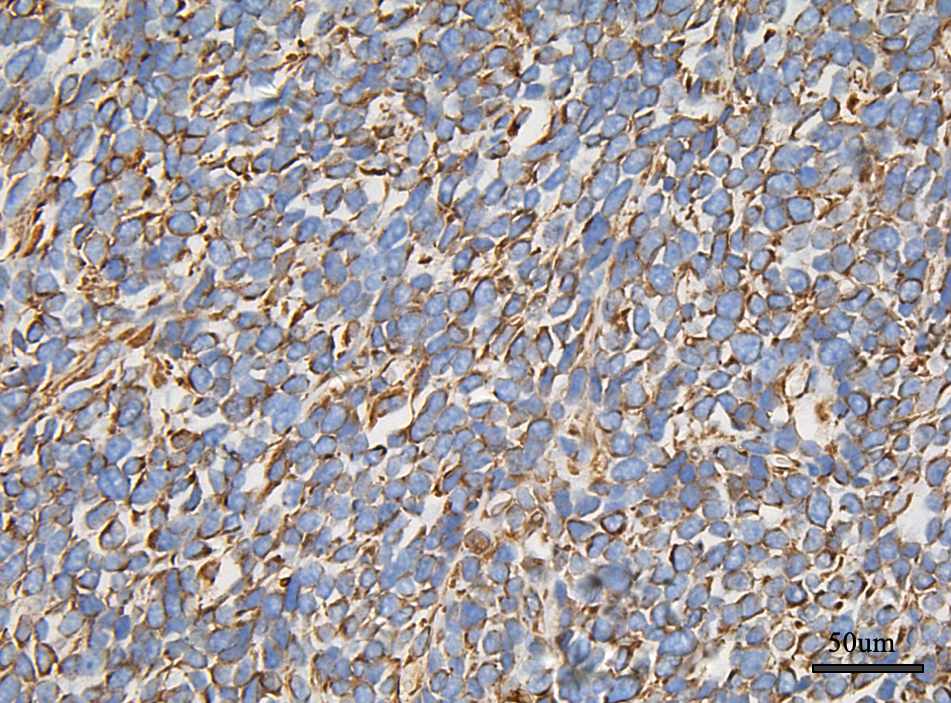


Immunohistochemistry suggested Vim（+）
